# Aspects of Toxoplasma Infection on the Reproductive System of Experimentally Infected Rams (*Ovis Aries*)

**DOI:** 10.1155/2009/602803

**Published:** 2009-05-27

**Authors:** Welber Daniel Zanetti Lopes, Alvimar José da Costa, Luis Fernando Santana, Ricardo Silva dos Santos, Walter Matheus Rossanese, Wilton Carlos Zanetti Lopes, Gustavo Henrique Nogueira Costa, Cláudio Alessandro Sakamoto, Thais Rabelo dos Santos

**Affiliations:** Department of Preventive Medicine, Animal Health Research Center (CPPAR), Faculty of Agricultural Sciences and Veterinary, São Paulo State University (UNESP), Via de acesso prof. Paulo Donatto Castellani, s/n CEP; 14884-900 Jaboticabal, SP, Brazil

## Abstract

Eight reproductive rams with no prior reproductive disease were distributed into three groups of infection with *T. gondii*: GI, 3 rams, 2.0 × 10^5^ P strain oocysts; GII, 3 rams, 1.0 × 10^6^ RH strain tachyzoites; GIII, 2 control rams. Clinical parameters were measured and serological evaluations (IIF) were performed. Presence of the parasite in the semen was investigated by PCR and bioassay techniques. The rams presented clinical alterations (hyperthermia and apathy) related to toxoplasmosis in both groups infected with *Toxoplasma gondii*. All the inoculated rams responded to antigenic stimulus, producing antibodies against *T. gondii* from postinoculation day 5 onwards. In ovine groups I and II, the greatest titers observed were 1 : 4096 and 1 : 8192, respectively. In semen samples collected from these two groups, the presence of *T. gondii* was detected by bioassay and PCR. This coccidian was isolated (bioassay and PCR) in tissue pools (testicles, epididymis, seminal vesicle, and prostrate) from two rams infected presenting oocysts and in one presenting tachyzoites.

## 1. Introduction

The *T. gondii* [1] is a feline intestinal coccidian with a variety of intermediate hosts among birds and mammals, including humans. In humans the disease was described by Junku (1923) and later detailed by [2]. Among others, [3] described the sexual cycle of *T. gondii* in felines. 

These animals, specially domestic cats, play a fundamental role in the transmission of toxoplasmosis, eliminating in the feces oocysts resulting from the sexual phase specific to the intestinal epithelium. The remaining hosts, including humans, only maintain the asexual phase of the cycle and serve as intermediate hosts [4]. 

Toxoplasmosis in ovine was described for the first time by [5], in the United States, in an ewe presenting nervous symptomology, increased temperature, and muscular rigidity. 

The high prevalence of toxoplasma infection in ovine could be linked to their low resistance to the parasite and to the fact that sheep raising procedures expose them to a greater probability of contact with oocysts eliminated by felines [6]. Toxoplasmosis has regularly been indicated as one of the major causes of abortions in ovine in several countries, including UK, New Zealand, the former USSR, Australia, India, and Canada [7–11]. Reproductive disorders, such as abortions, stillborn, or weak neonates that result in death, cause considerable economic losses to sheep flocks [12]. Studies conducted in Uruguay indicated toxoplasmosis as an important problem in sheep flocks with annual losses from 1.4 to 1.7 US$ million [13]. 

Another relevant aspect of this zoonose is the reporting of viable *T. gondii* cysts isolated from food tissue [10, 14]. Reference [15] recovered this coccidian from the brain and the diaphragm of 34 out of 40 sheep that were serologically positive by indirect immunofluorescence (IIF). The enormous losses caused by this disease, together with studies involving the isolation of viable parasites in food tissues, reveal the importance of ovine toxoplasmosis as a disease and as a zoonose.

Only three works in literature [16–18] report the isolation of *T. gondii* in ram semen. Complementing the research of those authors, the present study used two methods (bioassay and PCR) to isolate this coccidian in ram semen.

## 2. Material and Methods

P [19] and RH *T. gondii* strains [20] kept at the *Centro de Pesquisas em Sanidade Animal*, CPPAR, *Faculdade de Ciências Agrárias e Veterinárias*, FCAV, Universidade Estadual Paulista, UNESP were used. The inoculates were obtained through periodic inoculations of brain cysts (P strain) and/or tachyzoites (RH strain) in albino mice. *T. gondii* oocysts were obtained using a technique similar to that described by [21].

In this study, all procedures using animals complied with the Ethical Principles in Animal Research adopted by the College of Animal Experimentation (COBEA) and were approved by the Ethical Committee for Animal Welfare, UNESP, Jaboticabal, São Paulo, (CEBEA).

Eight rams, 13 to 14 months old, serologically negative for *T. gondii*, were selected, identified, randomized, and inoculated according to the experimental outline described in Table 1. The rams were maintained in individual bays belonging to CPPAR, with water and feed provided ad libitum. Serological exams to detect antibodies against other infectious diseases that could provoke reproductive disorders (brucellosis, neosporosis, and leptospirosis) were conducted on all the experimental rams, before and after inoculation. 

Antibodies against *T. gondii* were investigated by IIF. Serum samples were collected from all experimental rams two days before inoculation and on postinoculation days (PIDs) 3, 5, 7, 11, 14, and weekly thereafter until the end of the trial [22]. Concurrently, semen samples from all rams were obtained by use of an electroejaculator. Detection of *T. gondii* in the semen was carried out by bioassay [17] and by Polymerase Chain Reaction (PCR) [23].

In the bioassay, an aliquot of approximately 0.5 mL of semen (per ram) was inoculated in five mice, which were observed and examined according to the methodology adopted by [24]. The mice were considered positive for samples that showed a dilution >64 and the presence of *T. gondii* brain cysts of.

After the experimental period of semen collection (PID 70), all of the rams (inoculated groups and control) underwent postmortem examination in order to carry out further tissue parasitism evaluations by bioassay [25] and PCR [23]. 

DNA from semen samples, tissues, and positive controls (RH strain) was extracted for *T. gondii* detection [26]. Gene fragment B_1 _(194 bp) from *T. gondii* was thus amplified using primers 5^′^GGAACTGCATCCGTTCATGAG-3^′^ (B1_1_) and 5^′^TCTTTAAAGCGTTCGTGGTC-3^′^ (B1_2_), according to [23]. PCR carried out by the addition of 500 ng of genomic DNA in a reaction medium containing 2 mM MgCl_2_, 50 mM KCl, 10 mM Tris-HCl pH 9, 0.01% Triton X-100, 0.2 mM dNTPs, 10 pmoles of initiator, and 5.0 units of *Taq*DNA polymerase.

Analysis of the amplified products was done with the help of a 2% agarose gel containing restriction fragments separated by electrophoresis, stained with 0.5 *μ*g/mL of ethidium bromide solution dissolved in water for 20 minutes and observed by UV transilluminator.

## 3. Results

Toxoplasma infection in the reproductive rams used in this research was confirmed by seroconversion of the inoculated animals (Table 2). The mean body temperature of the control group under normal atmospheric conditions was 38°C. Considering that normal temperature in ovine oscillates between 37.5°C and 39.5°C [27], rectal temperature measurements revealed hyperthermia and anorexia in all the rams inoculated with oocysts (PID 5 and 7) and tachyzoites (PID 3, 5, and 7). 

**Figure 1:**
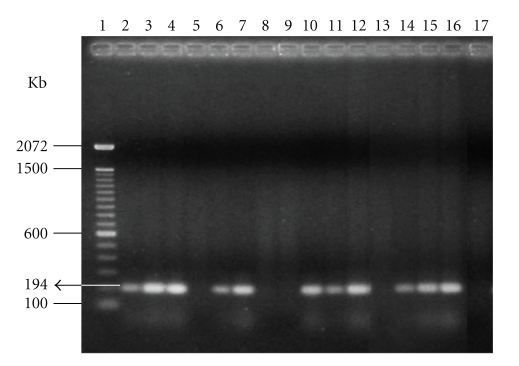
Electrophoresis in 2% agarose gel of PCR product extract from seminal samples of rams experimentally infected with *T. gondii*. (1) DNA Ladder, molecular weight marker (100 bp). (2) Ram 02 (PID 14). (3) Ram 09 (PID 35). (4) Ram 02 (PID 42). (5) Ram 16 (PID 49). (6) Ram 09 (PID 56). (7) Ram 02 (PID 63). (8) Ram 09 (PID 63). (9) Ram 07 (PID 5). (10) Ram 52 (PID 11). (11) Ram 48 (PID 14). (12) Ram 52 (PID 21). (13) Ram 52 (PID 49). (14) Ram 52 (PID 56). (15) Ram 07 (PID 70). (16) Positive control. (17) Negative control.

IgG class antibodies were detected by IIF from PID 5 onwards and remained at high levels until PID 56, when they diminished; however, none of the infected rams presented seronegative reactions by the end of the experiment (Table 2). Maximum serological titers (1 : 8192) were detected in a tachyzoite inoculated ram on PID 28. In all three oocyst-inoculated rams, the reciprocal serological maximum of 1 : 4096 was diagnosed on PID 49 and 56. Differences in reciprocal serological titers (*P* < .05) between the two inoculated groups (oocysts and tachyzoites) occurred on PID 11 and 14 (Table 2). The presence of *T. gondii* (bioassay) was detected in 14 ejaculates of the infected rams between PID 5 and 70 (Table 3). Of the seminal samples that proved positive in the bioassay (Table 3), *T. gondii* was diagnosed by PCR technique on PIDs 11, 14, 21, 56, and 70 in tachyzoite-inoculated animals and on PIDs 14, 35, 42, 56, and 63 in oocyst-inoculated animals (Figure 1).

**Figure 2:**
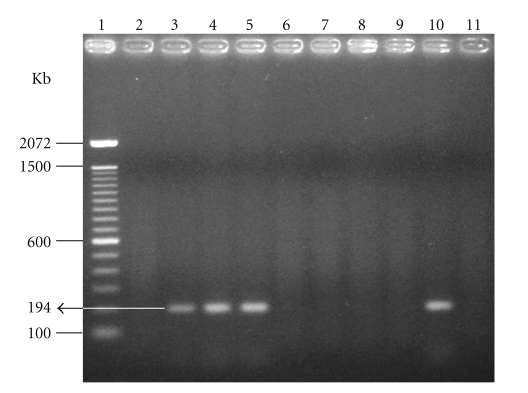
Electrophoresis in 2% agarose gel of PCR product extract from tissue pool samples (testicles, epididymis, seminal vesicle, and prostrate) of the experimental ram (1) DNA Ladder, molecular weight marker (100 bp). (2) Ram 02 (GI). (3) Ram 09 (GI). (4) Ram 16 (GI). (5) Ram 07 (GII). (6) Ram 48 (GII). (7) Ram 52 (GII). (8) Ram 43 (control). (9) Ram 44 (control). (10) Positive control. (11) Negative control.

In addition to seroconversion (IIF) of the animals and semen that proved positive for *T. gondii* (bioassay and PCR), it was also possible to isolate the coccidian (bioassay and PCR) in tissue pools (testicles, epididymis, seminal vesicle, and prostrate) of two oocyst-inoculated rams and one tachyzoite-inoculated ram (Figure 2).

## 4. Discussion

The results of the clinical parameters obtained in this research are similar to those found by other authors [28, 29]. Early humoral immune response in rams experimentally infected with *T. gondii* was also observed by [7].

This study reports the first description of the isolation of *T. gondii* from semen of experimentally infected rams using two diagnostic techniques: bioassay and PCR. In descriptions related to different strains, literature shows disagreement regarding the period this parasite can be recovered. [16] inoculated *T. gondii* in two rams and obtained infected semen (bioassay) on PID 20 from the first and on PID 25 from the second one. In another experiment, those authors systematically isolated *T. gondii* in two rams from PIDs 7 to 32 and from PIDs 14 to 32, respectively. 

Reference [17] isolated *T. gondii* (bioassay) from semen samples of three out of six inoculated rams, twice in each on PIDs 16 and 26. In Nigeria, using another strain (TS-I), [18] also isolated *T. gondii* in bioassay only from samples collected on PID 21 from all of the inoculated rams. The coccidian has also been isolated in seminal samples collected from goats [30], bovine [31], dogs [32], and swine [33].

In this study, a 194 bp fragment from the B_1_ gene of *T. gondii* was amplified because this gene was found to be highly conserved in several isolates and present in at least 35 loci of *T. gondii* [23, 34]. Similar to the results found by [31] in bovine, by [32] in dogs, and by [33] in swine, it should be highlighted that in the present work, the presence of *T. gondii* was only detected by the PCR technique in 10 of the 14 semen samples that were positive by bioassay. Given these results, it could be inferred that the bioassay (mouse inoculation) is more sensitive than PCR to the isolation of *T. gondii* in ram seminal samples (Table 3 and Figure 1). These inferences are reinforced by the results obtained by [35], who emphasized the superiority of the bioassay when compared to PCR for the isolation of *T. gondii*. [36] also confirmed this superiority, particularly regarding the isolation of this protozoan in ovine and bovine tissues. 

The absence of parasitism by PCR, as detected in some semen and tissue samples (Figures 1 and 2), does not rule out the presence of the parasitic agent. It is possible that part of the false negative results could be due to the DNA extraction technique, as well as the fact that 500 ng of genomic DNA (host + parasite) per reaction could contain an insufficiently quantity of parasite DNA to visualize the 194 bp fragment on 2% agarose gel [36–38]. For these reasons, certain authors affirm that PCR is a favorable method only in association with another means of diagnosis [39, 40]. 

These findings suggest another mode of infection for sheep thus perhaps contributing to the high prevalence of infection seen in this specie animal, once this infection immediately prior to could fuel a source of infection which might then lead to congenital infection or abortion [41, 42]. In summary this work showed the evidences in the aspects of infection by toxoplasmosis on the reproductive system of experimentally infected rams, however further study would be needed to investigate whether *T. gondii* could be sexually transmitted in flock of sheep.

## Figures and Tables

**Table 1:** Experimental outline of the rams inoculated with *T. gondii*.

Number of ovine	Group	*T.* *gondii* oocysts	*T.* *gondii* tachyzoites	Inoculation via
2		2 × 10^5^	—	Oral
9	I	2 × 10^5^	—	Oral
16		2 × 10^5^	—	Oral
7		—	1 × 10^6^	Subcutaneous
48	II	—	1 × 10^6^	Subcutaneous
52		—	1 × 10^6^	Subcutaneous
43	III	Placebo	Placebo	
44		Placebo	Placebo	

**Table 2:** Results of multiple comparisons and analysis of variance of the serological titers of noninoculated (control) and *T. gondii* oocyst-inoculated (2.0 × 10^5^) or tachyzoite-inoculated (1.0 × 10^6^) animals.

Postinoculation day	Reciprocal serological titers/mean* = ∑log(*x* + 1)/*n*		
	Control	Oocysts	Tachyzoites
−1	0.00_a_^A^	0.00_f_^A^	0.00_e_^A^
3	0.00_a_^A^	0.00_f_^A^	0.41_de_^A^
5	0.00_a_^B^	0.82_ef_^A^	1.01_d_^AB^
7	0.00_a_^B^	1.01_e_^A^	2.01_c_^A^
11	0.00_a_^C^	1.01_e_^B^	2.81_abc_^A^
14	0.00_a_^C^	1.71_de_^B^	2.61_bc_^A^
21	0.00_a_^C^	3.41_ab_^A^	3.01_ab_^B^
28	0.00_a_^B^	2.61_bcd_^A^	2.61_bc_^A^
35	0.00_a_^B^	2.71_abc_^A^	0.00_e_^A^
42	0.00_a_^B^	3.21_abc_^A^	3.21_ab_^A^
49	0.00_a_^B^	3.61_a_^A^	3.31_ab_^A^
56	0.00_a_^B^	3.61_a_^A^	3.61_a_^A^
63	0.00_a_^B^	2.40_cd_^A^	2.61_bc_^A^
70	0.00_a_^B^	2.51_bcd_^A^	2.71_abc_^A^

*F* value and significance	Análise de variância		
	Groups		147.59**
	period		53.85**
	groups X period		15.09**

*Means followed by the same letter, capitals in the columns and small along the lines, do not differ between each other by the Tukey test (*P* > .05).

**Table 3:** Investigation of *T. gondii* in seminal samples of noninoculated (control) and *T. gondii* oocyst-inoculated (2.0 × 10^5^) or tachyzoite-inoculated (1.0 × 10^6^) animals.

Ovine *N*°	Inoculate	Postinoculation day														
		−1	3	5	7	11	14	21	28	35	42	49	56	63	70	Total
43	Control	—	—	—	—	—	—	—	—	—	—	—	—	—	—	0
44		—	—	—	—	—	—	—	—	—	—	—	—	—	—	0

Total		0	0	0	0	0	0	0	0	0	0	0	0	0	0	0

7	Tachyzoites	—	—	Positive*	—	—	—	—	—	—	—	NR	—	—	Positive*	2
48		—	—	—	—	—	Positive*	—	—	—	—	—	—	—	—	1
52		—	—	—	—	Positive*	—	Positive	—	—	—	Positive*	Positive*	—	—	4

Total		0	0	1	0	1	1	1	0	0	0	1	1	0	0	7

2	Oocysts	—	—	—	—	—	Positive*	—	—	—	Positive	—	—	Positive	—	3
9		—	—	—	—	—	—	—	—	Positive*	—	—	Positive*	Positive	—	3
16		—	—	—	—	—	—	—	—	—	—	Positive*	—	—	—	1

Total		0	0	0	0	0	1	0	0	1	1	0	1	1	0	7

Positive = IIF for *T. gondii* (≥1 : 64) and presence of brain cysts in inoculated mice.

Positive* = presence of brain cysts in inoculated mice and by PCR technique.

— = Negative serology (mice).

NR = Not realized (insufficient seminal sample).
